# Mr. Franks, on the Brunonian System

**Published:** 1799-04

**Authors:** John Franks


### *To the Editors of the Medical and Phyſical Journal.*

GENTLEMEN, *Great Smith-Street, Weſtminſter, March* 8, 1799.

AS the Medical and Phyſical Journal is intended to be conducted on a broad and impartial baſis, I hope you will inſert in your next number the following obſervations, on a ſubject which I conceive to be of great importance, and which merits the moſt ſerious conſideration of every medical practitioner,

Gentlemen, with the greateſt reſpect

I am your moſt obedient Servant,

JOHN FRANKS.

In the firſt number of your Journal we find an article extracted from an eſſay written by Profeſſor HUFELAND, of Jena, intitled "Remarks on the influence of the Brunonian ſyſtem on the practice of medicine."

There

There are Phyficians in this Country, who entertain the fame opinion with Profeffor Hufeland, that Doctor Brown's work has not a tendency to facilitate the acquifition of medical knowledge, or to improve the method of curing difeafes. On the other hand, there are a very great number of medical practitioners of great refpectability, who from their own experience of its utility, have adopted a very different opinion, amongft thefe are Dr. Blane, Dr. Robertson, and Dr. Trotter.

We find the fame fort of unfubftantial objections now begun to be made to this fyftem of medicine abroad, as were made to it ten years ago at home.

To fatisfy the minds of young men who have not yet commenced their medical career, fomething more is neceffary in order to convince them, that this doctrine is not the model they ought to take as their guide, than mere general affertions, witticifms, a reference to the difficulties of applying it in practice, and a contemptuous manner of treating the memory of the Author.

"That animal life is a forced ftate of exiftence, is a fact, fays a Reviewer, which has probably been known ever fince eating and drinking were in fafhion."—Granted.—But has any practical application ever been made of it, we would afk? Has it not been referved for Dr. Brown to make a practical application of this fact, and with the affiftance of a multitude of others, to eftablifh a doctrine, which will probably be well received by the lateft pofterity.

Believing, as I do, the vaft importance of this doctrine to individuals and to the ftate, from the great number of lives which have been faved this war by an application of its principles, I wifh to fee the whole matter fairly prefented to the public, becaufe I think the refult will be, that the truth of it will be more univerfally acknowledged.

It may be, and no doubt is, in practice fometimes very difficult to diftinguifh a fthenic difeafe from a difeafe of the afthenic clafs; yet if this divifion of difeafes is found to be natural, thefe difficulties ought not to be afcribed to the doctrine, but to the imperfect ftate of human knowledge.

But although it may fometimes occur in practice, that from the equivocal nature of fymptoms we fhould be deceived; the indications of cure being applied to the cafe, will foon point out the error into which we have been precipitated by fallacious appearances, and will be remedied by reverfing the mode of treatment; for it is not to be fuppofed, that a prudent practitioner would immediately have recourfe to the moft powerful means of removing a diatheſis of an uncertain nature. Thoſe who are thoroughly acquainted with the Brunonian ſyſtem, cannot act ſyſtematically wrong, and this is more perhaps, than can be pleaded in behalf of any other ſyſtem. Although on our firſt viſit to a patient, it is true, that we have only to revolve in our minds ſuch queries as theſe: Is this diſeaſe ſthenic or aſthenic? is it univerſal or local? next, what is its degree? yet, we ſhall not, in every caſe, be able to determine theſe queries, ſo eſſentially neceſſary to be known; on the contrary, in many caſes in practice, it will require the greateſt exertion of judgment, to form a right concluſion.

But a right concluſion muſt be formed, or we cannot cure the diſeaſe, and an application of our teſt in theſe caſes, will remove the difficulty, for "the powers that produce one claſs of diſeaſes, are the remedies for the other, and *vice verſa.*"

Dr. Brown's ſyſtem of medicine is founded on uncontroverted facts, and philoſophical induction, and not like thoſe ſyſtems which preceded it, on fanciful hypotheſis; therefore, will ſeldom miſlead us in practice. The doctrine in general is plain, ſimple, and eaſy to be comprehended. In it we obſerve only a few general diſtinctions, correſponding to the differences really found to exiſt. In other works of this ſort, we find a great number of diſtinctions made between diſeaſes not eſſentially differing in kind. If we are governed in our practice by ingenious theories which have no foundation in nature, caſes would frequently occur, where, on account of the ſymptoms, contra-indications of cure would be ſuppoſed neceſſary. According to this work, although in many caſes, ſecondary or partial conſiderations may preſent themſelves, yet the principal indications in every univerſal diſeaſe are the ſame, namely, to *ſtimulate* or *debilitate,* and to apportion our remedies to the degree of the diſeaſe, and not to ſtimulate on account of certain ſymptoms, and debilitate on account of others, or continue the practice of ſtimulating and debilitating alternately, until the death of the patient puts a period to our endeavours.

Our author has clearly demonſtrated to us, the real nature of animal life, has ſhewn that every diſeaſe of the ſyſtem is preceded by prediſpoſition; that health, pre-diſpoſition, and general diſeaſe, are ſimilar, and not *appoſite* ſtates of the body, differing according to the *degree of excitement only.* "The excitement is every thing, ourſelves nothing; for no longer excitement, no longer life." Again, "if a juſt degree of excitement could be conſtantly kept up, mankind would enjoy *eternal health.*" If a certain degree of excitement is the cauſe of health, it neceſſarily follows, that the application of a leſs or a greater degree of this *effect,* immediately places the the body either below or above the healthy ſtate; and conſequently, a pre-diſpoſition to a diſeaſe of the aſthenic, or ſthenic claſſes directly commences, and which will, ſooner or later, terminate in actual diſeaſe, unleſs counter-acted by an increaſe or diminution of the excitement. During the period of prediſpoſition, which may be of longer or ſhorter date; for the difference in the degree of the *effect*, compenſates for the difference of time; ſo little diſturbance in the animal economy is noticed, that on the appearance of the ſymptoms of diſeaſe, it would ſeem as if it had ſuddenly invaded the human conſtitution, but which, this doctrine ſhews, is not the caſe. From the increaſe, or diminution, of excitement, a pre-diſpoſition to diſeaſe is formed, and is gradually, but at the ſame time imperceptibly, increaſing; until, as it may often happen, an occaſional cauſe is applied, which, with the exiſting pre-diſpoſition, induces diſeaſes that have often been aſcribed to a *cauſe* which has not evidently operated ſo as to induce them; the cauſe here alluded to, is *contagion*. On this ſubject it may not be improper, perhaps, to offer a few remarks. A medical friend, who is an able advocate for the Brunonian doctrine, has taken notice of a circumſtance, which appears to demand attention, for at firſt ſight, it appeared to him, rather to militate againſt it.—It is contagion. "I admire the univerſal doctrine of ſtimuli," obſerves this gentleman, "nor do I find it, in the leaſt, vulnerable, except in the conſideration of contagion; in this reſpect, Brown is unſatisfactory. A reference to the general propoſition is not ſufficient." He then aſks, "What is contagion? a poſitive or negative principle? or does not fever, ariſing from the ſuppoſed introduction of this *unknown* power, rather depend, where there is no other evident remote cauſe, as cold, fear, famine, &c. upon impurity of the air? The air is a ſtimulus abſolutely neceſſary to life; its ſtimulant degree depends upon its purity, any thing diminiſhing *this*, and rendering it not ſufficiently ſtimulating, may give occaſion to every circumſtance of the febrile ſtate; this idea leads me, at once, to diſclaim the notion of a poſitive ſedative, and to embrace the Brunonian opinion, that all diſeaſes of direct debility ariſe from an abſtraction of neceſſary ſtimuli".—I have extracted theſe few lines from a very long letter on the ſubject, to ſhew, that the advocates for the doctrine have not given it a decided preference, without due examination.

The ſcience of medicine has, in many inſtances, been improved by empirics, but this conſideration is not a ſufficient reaſon that we ſhould act empirically on every occaſion, becauſe on a few, we are obliged to content ourſelves with obſerving effects, without knowing in what manner thoſe effects have been induced. We have long known, that mercury cures the venereal diſeaſe; we were obliged to remain ſatiſfied with the fact, fact. Now that we know, or are ſuppoſed to know, upon what principle it is, that this remedy produces its effects, ſubſtitutes that poſſeſs a leſs dangerous tendency, are aſſiduouſly enquired for.

It is therefore abſolutely neceſſary, that we ſhould be in poſſeſſion of ſome ſyſtem of medicine. If Dr. Brown's ſyſtem is only in the ſmalleſt degree preferable to thoſe which have preceded it, the work deſerves attention. It may have defects, but thoſe defects ought to be conſidered as ſo many exceptions: and exceptions were never yet conſidered of ſuch importance as to deſtroy the propriety of general principles.

The aphoriſms of Brown are ſo intimately connected, originating from known facts, and philoſophical induction, that they appear to me likely to ſtand the teſt of ages; and I think that had his life been extended a few years, the healing art, before his time ſo myſterious, conjectural, and contradictory, would have received from his labours ſtill further elucidation.

